# Supplementary material for: Characterisation of ethnic differences in DNA methylation between UK-resident South Asians and Europeans
Source: Clin Epigenetics. 2022 Oct 15;14:130. doi: 10.1186/s13148-022-01351-2 (PMC9571473; doi:10.1186/s13148-022-01351-2)
Supplement: Supplementary file 1 — Additional file 1. Figure S1. Principal component analysis of SABRE genetic data. The upper panels show PCs 1 and 2 generated from SABRE or BiB data. The lower panels show PCs 1 and 2 generated from SABRE + HapMap3 data or BiB + HapMap3 data. Colours indicate self-reported ethnic group or subgroup (SABRE, BiB) or population group (HapMap3). Axis labels show the variance explained by each PC. HapMap3 populations have been collapsed: South Asian = GIH; European = CEU + TSI; African = ASW + LWK + MKK; Mexican = MEX; South East Asian = CHB + CHD + JPT. In the lower panel (SABRE), two outliers self-reporting as South Asian in SABRE data appear intermediate between African groups from HapMap3 and the remaining South Asian cluster. Both of these individuals reported their country of birth as an African country indicating possible genetic admixture in these individuals. These two individuals were removed from all other analyses. In the lower panel (SABRE), “other South Asian” individuals predominantly identify their country of birth as South Asia (India, Pakistan, Bangladesh, Sri Lanka), n = 42/50. [file 13148_2022_1351_MOESM1_ESM.pdf]

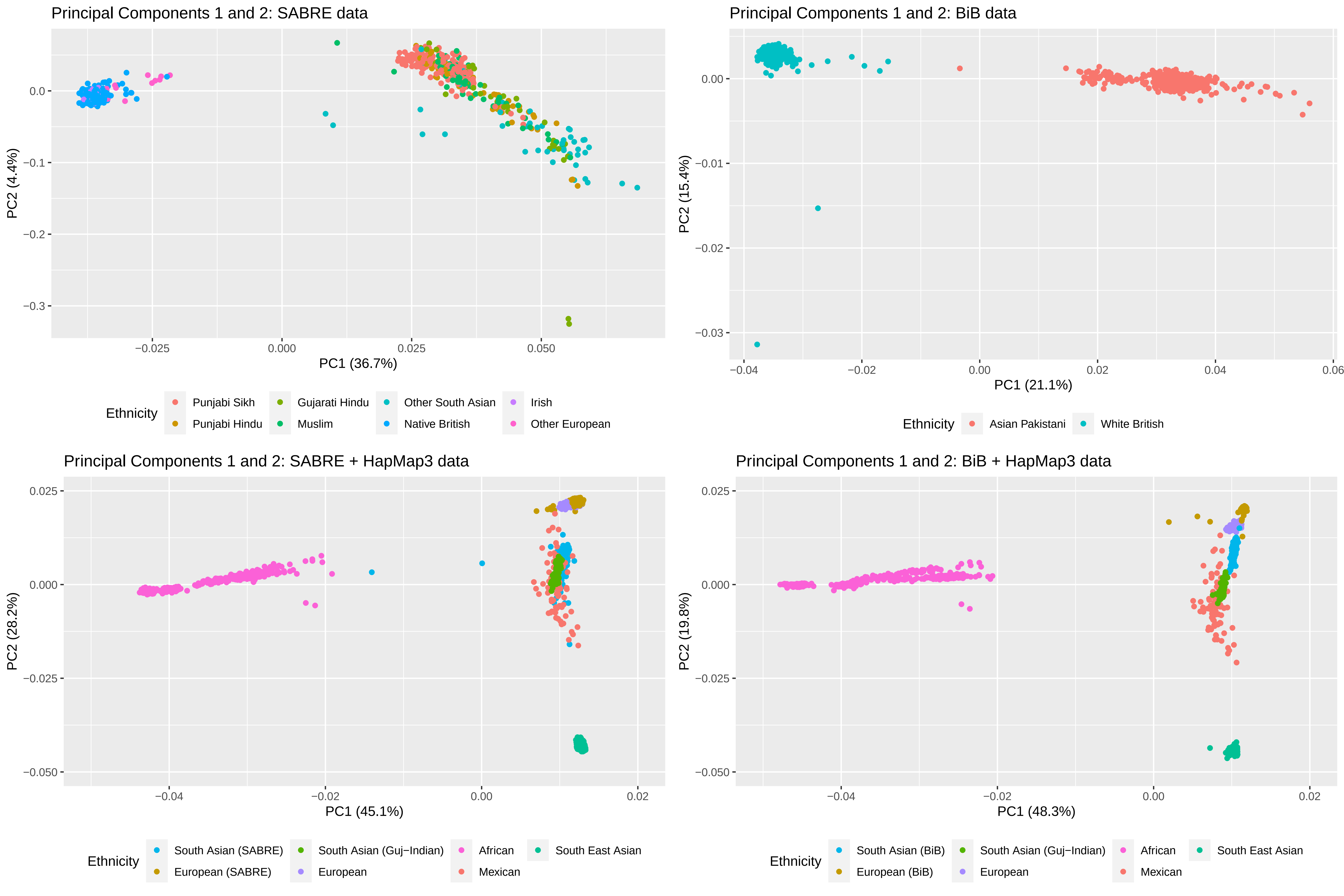
Principal Components 1 and 2: SABRE data
PC2 (4.4%)
0.0
−0.1
−0.2
−0.3
−0.025
0.000
0.025
0.050
PC1 (36.7%)
Ethnicity
Punjabi Sikh
Punjabi Hindu
Gujarati Hindu
Muslim
Other South Asian
Native British
Irish
Other European
Principal Components 1 and 2: BiB data
PC2 (15.4%)
0.00
−0.01
−0.02
−0.03
−0.04
−0.02
0.00
0.02
0.04
0.06
PC1 (21.1%)
Ethnicity
Asian Pakistani
White British
Principal Components 1 and 2: SABRE + HapMap3 data
PC2 (28.2%)
0.025
0.000
−0.025
−0.050
−0.04
−0.02
0.00
0.02
PC1 (45.1%)
Ethnicity
South Asian (SABRE)
European (SABRE)
South Asian (Guj−Indian)
European
African
Mexican
South East Asian
Principal Components 1 and 2: BiB + HapMap3 data
PC2 (19.8%)
0.025
0.000
−0.025
−0.050
−0.04
−0.02
0.00
0.02
PC1 (48.3%)
Ethnicity
South Asian (BiB)
European (BiB)
South Asian (Guj−Indian)
European
African
Mexican
South East Asian
