# Supplementary material for: Characterisation of ethnic differences in DNA methylation between UK-resident South Asians and Europeans
Source: Clin Epigenetics. 2022 Oct 15;14:130. doi: 10.1186/s13148-022-01351-2 (PMC9571473; doi:10.1186/s13148-022-01351-2)
Supplement: Supplementary file 2 — Additional file 2. Figure S2. Principal component analysis of SABRE DNA methylation data. The upper panel shows PCs 1 and 2 generated from the 1000 most variant probes in SABRE or BiB. The lower panel shows PCs 1 and 2 generated from the 10000 most variant probes in SABRE or BiB. Colours indicate self-reported ethnic subgrouping (SABRE, BiB). Axis labels show the variance explained by each PC. [file 13148_2022_1351_MOESM2_ESM.pdf]

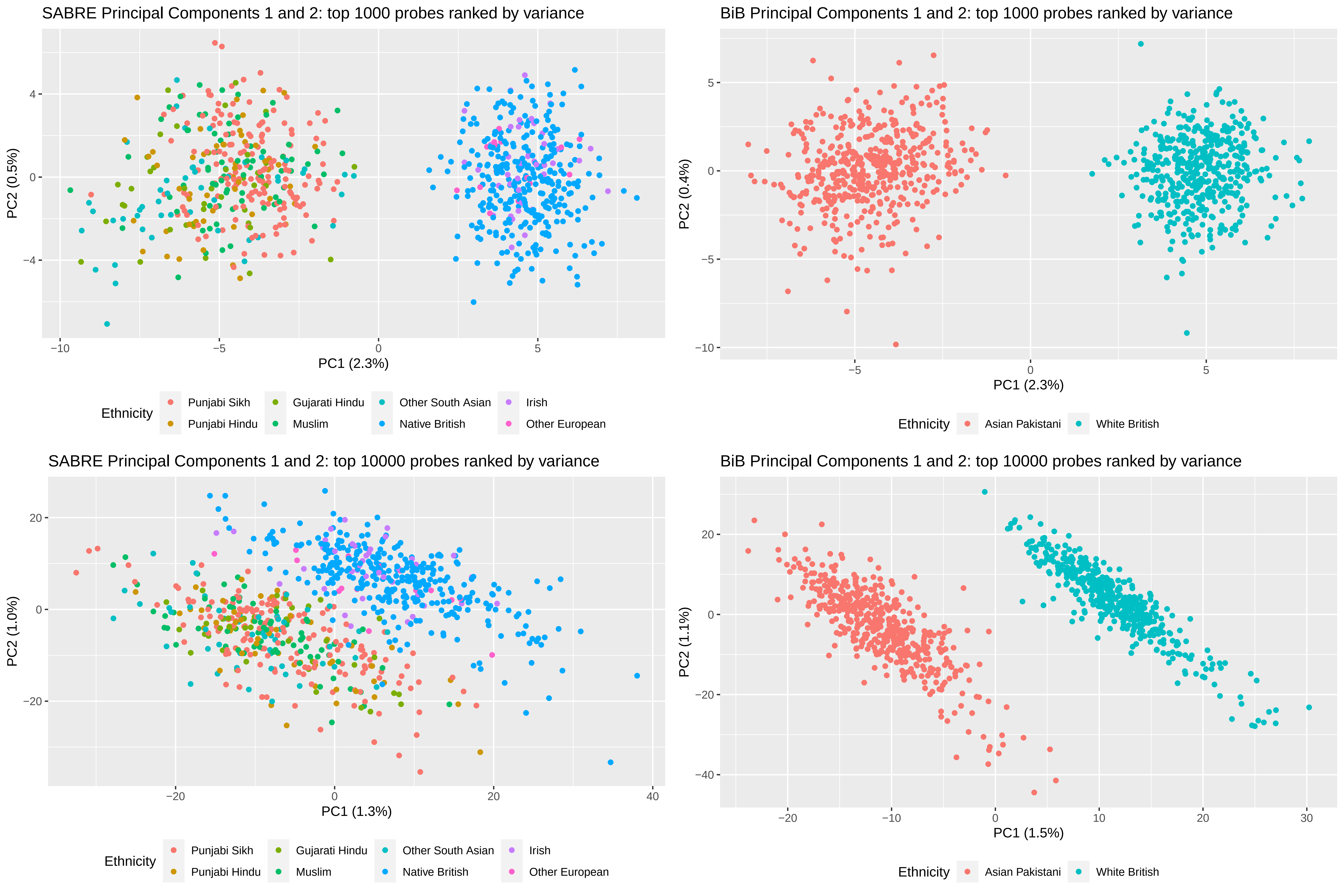

SABRE Principal Components 1 and 2: top 1000 probes ranked by variance
PC2 (0.5%)
PC1 (2.3%)
4
0
−4
−10
−5
0
5
Ethnicity
Punjabi Sikh
Punjabi Hindu
Gujarati Hindu
Muslim
Other South Asian
Native British
Irish
Other European
BiB Principal Components 1 and 2: top 1000 probes ranked by variance
PC2 (0.4%)
PC1 (2.3%)
5
0
−5
−10
−5
0
5
Ethnicity
Asian Pakistani
White British
SABRE Principal Components 1 and 2: top 10000 probes ranked by variance
PC2 (1.0%)
PC1 (1.3%)
20
0
−20
−20
0
20
40
Ethnicity
Punjabi Sikh
Punjabi Hindu
Gujarati Hindu
Muslim
Other South Asian
Native British
Irish
Other European
BiB Principal Components 1 and 2: top 10000 probes ranked by variance
PC2 (1.1%)
PC1 (1.5%)
20
0
−20
−40
−20
−10
0
10
20
30
Ethnicity
Asian Pakistani
White British
