# Supplementary figures and images for: Characterisation of ethnic differences in DNA methylation between UK-resident South Asians and Europeans
Source: Clin Epigenetics. 2022 Oct 15;14:130. doi: 10.1186/s13148-022-01351-2 (PMC9571473; doi:10.1186/s13148-022-01351-2)

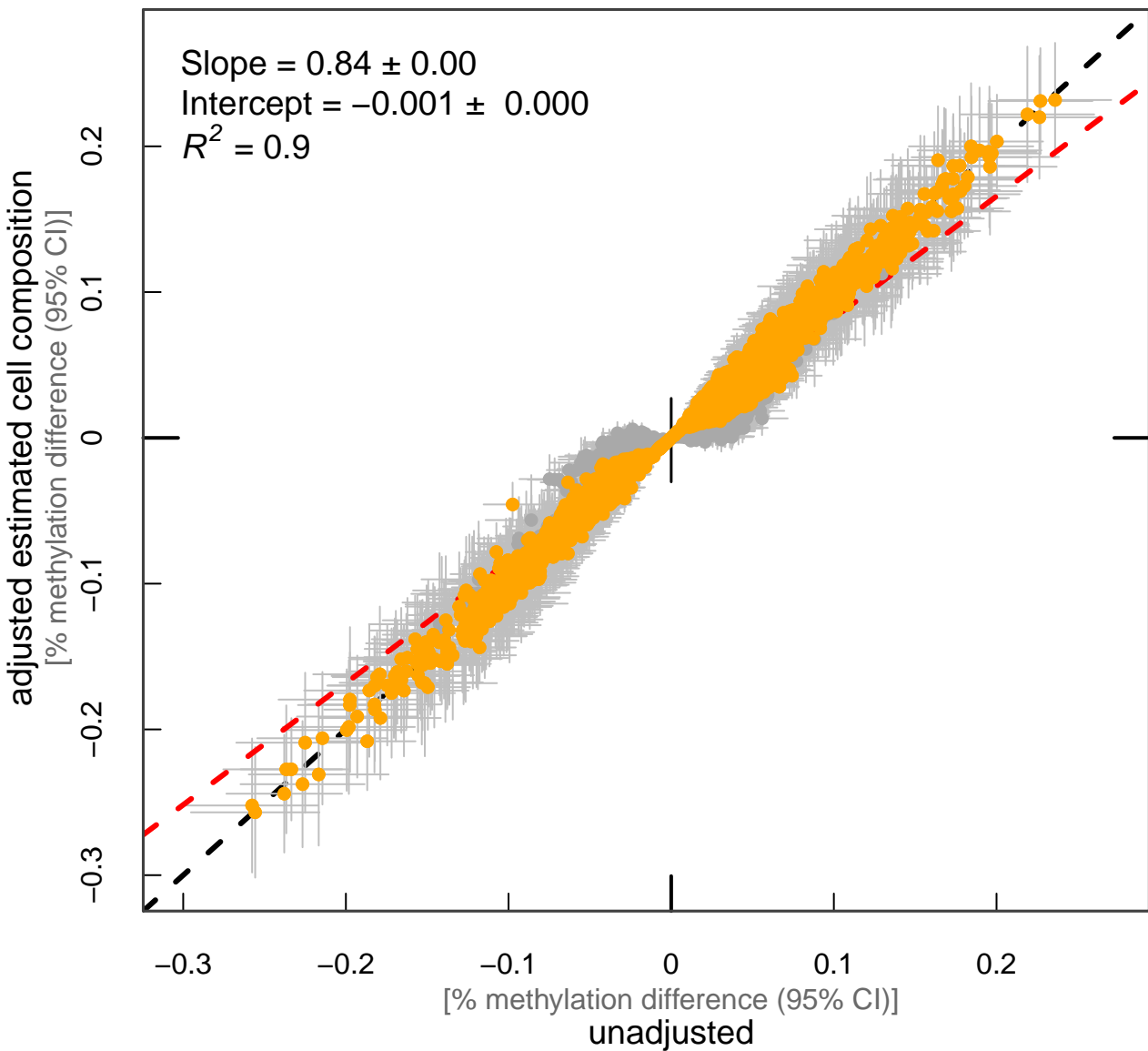

Supplement: Supplementary file 5 — Additional file 5. Figure S3. Comparison of effect sizes for cell composition adjustment of EWAS. Each CpG is represented by a point on the graph with 95% confidence intervals for effect estimates. Red dashed line: linear regression between data sets. Black dashed line: line of equality. Orange highlighted estimates: p ≤ 1.03 × 10^−7^ cell adjusted EWAS (n = 3922/16,344 CpG sites). [file 13148_2022_1351_MOESM5_ESM.pdf]
